# Role of *VEGF* Polymorphisms in the Susceptibility and Severity of Interstitial Lung Disease

**DOI:** 10.3390/biomedicines9050458

**Published:** 2021-04-22

**Authors:** Sara Remuzgo-Martínez, Fernanda Genre, Verónica Pulito-Cueto, Belén Atienza-Mateo, Víctor Manuel Mora Cuesta, David Iturbe Fernández, Sonia María Fernández Rozas, Leticia Lera-Gómez, Pilar Alonso Lecue, María Piedad Ussetti, Rosalía Laporta, Cristina Berastegui, Amparo Solé, Virginia Pérez, Alicia De Pablo Gafas, Oreste Gualillo, José Manuel Cifrián, Raquel López-Mejías, Miguel Ángel González-Gay

**Affiliations:** 1 Genetic epidemiology and Atherosclerosis in Systemic Diseases and in Metabolic Bone Diseases of the Musculoskeletal System, IDIVAL, 39011 Santander, Spain; sara.r.mtz@gmail.com (S.R.-M.); fernandagenre@gmail.com (F.G.); veronica_pulito_cueto@hotmail.com (V.P.-C.); mateoatienzabelen@gmail.com (B.A.-M.); victormanuel.mora@scsalud.es (V.M.M.C.); diturfer@gmail.com (D.I.F.); soniam.fernandez@scsalud.es (S.M.F.R.); letizialera@hotmail.com (L.L.-G.); alonsolecue@hotmail.com (P.A.L.); josemanuel.cifrian@scsalud.es (J.M.C.); 2 López Albo’ Post-Residency Programme, Hospital Universitario Marqués de Valdecilla, 39008 Santander, Spain; 3 Rheumatology Department, Hospital Universitario Marqués de Valdecilla, 39008 Santander, Spain; 4 Pneumology Department, Hospital Universitario Marqués de Valdecilla, 39008 Santander, Spain; 5 Pneumology Department, Hospital Universitario Puerta de Hierro, 28222 Majadahonda, Spain; mariapiedad.ussetti@salud.madrid.org (M.P.U.); roslaporta@gmail.com (R.L.); 6 Pneumology Department, Hospital Universitario Vall d’Hebron, Universidad Autónoma de Barcelona, 08035 Barcelona, Spain; cberastegui@vhebron.net; 7 Lung Transplant and Cystic Fibrosis Unit, Hospital Universitario y Politécnico La Fe, 46026 Valencia, Spain; sole_amp@gva.es; 8 Lung Transplant Unit, Division of Pulmonology, Hospital Universitario 12 de Octubre, 28041 Madrid, Spain; vluz71@hotmail.com (V.P.); alic1575@separ.es (A.D.P.G.); 9 SERGAS (Servizo Galego de Saude) and IDIS (Instituto de Investigación Sanitaria de Santiago), NEIRID Lab. (Neuroendocrine Interactions in Rheumatology and Inflammatory Diseases), Research Laboratory 9, Santiago University Clinical Hospital, 15706 Santiago de Compostela, Spain; Oreste.Gualillo@sergas.es; 10 School of Medicine, Universidad de Cantabria, 39011 Santander, Spain; 11 Cardiovascular Pathophysiology and Genomics Research Unit, School of Physiology, Faculty of Health Sciences, University of the Witwatersrand, 2000 Johannesburg, South Africa

**Keywords:** interstitial lung disease, idiopathic interstitial pneumonia, vascular endothelial growth factor, biomarker, genetics

## Abstract

The search for biomarkers that can help to establish an early diagnosis and prognosis of interstitial lung disease (ILD) is of potential interest. *VEGF* polymorphisms have been implicated in the development of several lung disorders. Consequently, we assessed, for the first time, the role of *VEGF* polymorphisms in the susceptibility and severity of ILD. A total of 436 Caucasian ILD patients (244 with idiopathic interstitial pneumonias (IIPs) and 192 with non-IIP) and 536 ethnically-matched healthy controls were genotyped for *VEGF* rs833061, rs1570360, rs2010963, rs3025020, and rs3025039 polymorphisms by TaqMan assays. Pulmonary function tests were collected from all the patients. VEGF serum levels were determined by ELISA in a subgroup of patients. No *VEGF* genotype, allele, carrier, or haplotype differences were found between ILD patients and controls as well as between IIP and non-IIP patients. However, an association of rs1570360 with IIP in women and also with lung function in IIP patients was found. None of the *VEGF* polymorphisms were associated with VEGF levels. In conclusion, our results suggest that *VEGF* does not seem to play a relevant role in ILD, although rs1570360 may influence the severity of ILD in women and a worse outcome in IIP patients.

## 1. Introduction

The term interstitial lung disease (ILD) comprises a large group of diseases caused by chronic inflammation and fibrosis of the alveoli wall that share similar functional, clinical, radiological, and anatomopathological characteristics. ILDs can be classified according to their cause into idiopathic interstitial pneumonias (IIPs) or known cause ILDs [1,2,3]. Idiopathic pulmonary fibrosis (IPF) is the most frequent and severe IIP, characterized by a progressive dyspnea along with respiratory failure and a poor prognosis [4]. It is well known that the etiology of ILD is complex, and besides internal and external risk factors, a genetic component contributes to the development and severity of ILD [5]. The accurate diagnosis of these ILDs supposes a challenge for the clinicians and a multidisciplinary approach is frequently necessary [6].

The study of potential blood biomarkers in the pathogenesis of ILD is considered to be of potential interest, since they may be used as additional tools for the early diagnosis and severity prognosis of these pathologies. Furthermore, they are more reproducible and easier to obtain than conventional invasive methods of diagnosis [7,8,9].

Vascular endothelial growth factor (VEGF) is a tyrosine kinase glycoprotein that plays a key role as a mediator of angiogenesis and vasculogenesis in both physiological and pathological processes, being implicated in numerous inflammatory and chronic conditions [10,11,12,13,14,15,16,17,18]. In particular, it is well known that VEGF contributes to the normal lung maintenance, repair of pulmonary circulation, and modulation of the angiogenic process mainly related to endothelial cells [19,20,21]. In this line, some *VEGF* functional polymorphisms have been shown to influence the susceptibility to several lung disorders such as pulmonary hypertension and chronic obstructive pulmonary disease (COPD), among others [22,23,24,25,26]. However, studies on the genetic role of *VEGF* in ILD are scarce although VEGF protein levels have been proposed as a potential prognostic biomarker in IPF [7,18,27,28]. In fact, nintedanib, a tyrosine kinase inhibitor of VEGF receptors along with other signaling pathways, is one of the main drugs approved for the treatment of IPF [29].

Given that lung inflammation, altered fibrosis, and aberrant angiogenesis are typical processes in ILD pathogenesis, it is plausible to think that *VEGF* is implicated in ILD. Therefore, in this study we aimed to assess, for the first time, the role of *VEGF* polymorphisms in the susceptibility and severity of ILD in a large cohort of Caucasian patients with this disease.

## 2. Materials and Methods

### 2.1. Patients and Controls

A total of 436 Caucasian patients with ILD, fulfilling the American Thoracic Society/European Respiratory Society classification and diagnosis criteria for ILD [2,4,30] were recruited between May 2016 and December 2019 from the following referral Spanish hospitals: Hospital Universitario Marqués de Valdecilla (Santander), Hospital Puerta de Hierro and Hospital 12 de Octubre (Madrid), Hospital Universitario Vall d’Hebrón (Barcelona), and Hospital Universitario y Politécnico de la Fe (Valencia). Information on pulmonary function tests (PFTs) was collected from all the patients. Specifically, forced vital capacity (FVC), forced expiratory volume in the first second (FEV1), and diffusing capacity of the lungs for carbon monoxide (DLCO) were recorded at the time of recruitment. The whole cohort of patients with ILD was composed of 244 patients with IIP and 192 with known cause ILD (called non-IIP patients from now on). In particular, the group of IIP included mainly patients with IPF as well as patients with fibrotic idiopathic nonspecific interstitial pneumonia and unclassifiable IIPs, according to Cottin et al.’s classification [3]. The group of non-IIP was made up of the following categories: Patients with autoimmune ILDs, hypersensitivity pneumonitis, fibrosing pulmonary sarcoidosis, and other ILDs [3]. Demographic and clinical characteristics of the whole cohort of ILD patients, IIP patients, and non-IIP patients are shown in Table 1. In addition, the features of the non-IIP patients stratified according to the categories above mentioned are shown in Supplementary Table S1.

Five hundred and thirty-six ethnically-matched healthy controls, without a history of any pulmonary or chronic inflammatory disease, from Hospital Universitario Marqués de Valdecilla (Santander) and National DNA Bank Repository (Salamanca), were also included in this study.

All patients with ILD and healthy controls gave their informed consent to be included in the study. The procedures followed were in accordance with the ethical standards of the approved guidelines and regulations, according to the Declaration of Helsinki. All experimental protocols were approved by the local ethics committees of each participant hospital.

### 2.2. VEGF Polymorphisms Selection and Genotyping

*VEGF* polymorphisms were selected based on the following criteria: Polymorphisms with a frequency >5% in Caucasians previously assessed and reported to be involved in susceptibility to several inflammatory and pulmonary diseases [13,15,22,23,24,25,31]; functional polymorphisms associated with VEGF protein production [13,32]; and polymorphisms distributed along the gene. Accordingly, *VEGF* rs833061 and rs1570360 located in the *VEGF* promoter region, rs2010963 located in 5′untranslated region (UTR), rs3025020 located in intron 6, and rs3025039 located in 3′UTR of the *VEGF* gene were chosen.

Genomic DNA was extracted from peripheral blood using the REALPURE “SSS” kit (RBME04, REAL, Durviz S.L., Valencia, Spain). The quality and quantity of extracted DNA was measured in a spectrophotometer (NanoDrop ND-1000, Wilmington, DE, USA). All patients and healthy controls were genotyped for *VEGF* rs833061 T/C, rs1570360 G/A, rs2010963 G/C, rs3025020 C/T, and rs3025039 C/T polymorphisms by TaqMan assays. Negative controls and duplicate samples were included to check the accuracy of the genotyping. Genotyping was performed in a QuantStudioTM 7 Flex real-time polymerase chain reaction system (Applied Biosystems, Foster City, CA, USA).

### 2.3. Serum VEGF Determination

VEGF serum levels were determined in a subgroup of 272 ILD patients (133 IIP and 139 non-IIP) by a commercial ELISA kit (Reddot Biotech Inc., Kelowna, BC, Canada) in accordance with the manufacturer’s instructions. Samples were analyzed in duplicate. VEGF levels were quantified using a four-parameter logistic curve fit suitable for calculating concentrations from symmetrical sigmoidal calibrators through MyAssays software.

### 2.4. Statistical Analysis

Data were reported as the number of individuals (n) and percentage (%) for categorical variables and mean ± standard deviation (SD) for continuous variables. All genotype data were checked for deviation from Hardy–Weinberg equilibrium (HWE). For the estimation of allele frequencies, the number of individuals was duplicated considering that each individual carries 2 alleles of each polymorphism (one allele in each chromosome). Differences in *VEGF* genotypes, alleles, and carriers of the minor allele frequencies between the whole group of ILD patients and controls were calculated and compared by a chi-square test. Similarly, genetic differences between IIP patients and the whole group of non-IIP patients as well as between IIP patients and the different categories of the group of non-IIP patients (autoimmune ILDs, hypersensitivity pneumonitis, other ILDs and sarcoidosis) were assessed. Additionally, differences in the frequency of *VEGF* polymorphisms were analyzed between ILD patients and controls as well as between the group of patients with IIP and non-IIP patients, stratifying the population according to sex. Haplotype frequencies were calculated by the Haploview 4.2 software and then, compared between the groups mentioned above by a chi-square test. Strength of associations was estimated using odds ratios (OR) and 95% confidence intervals (CI). OR and *p*-values were adjusted by sex, age, smoking history, and packs of cigarettes per year when genetic differences between IIP and non-IIP patients were evaluated. Analyses regarding the association between *VEGF* and PFTs or VEGF serum levels in IIP and non-IIP patients were performed with carriers, given that the frequency of the TT genotype of rs3025039 was lower than 5%. These associations were assessed by linear regression, adjusting *p*-values for the potential confounding factors previously mentioned. In addition, to account for the five polymorphisms assessed, the Bonferroni adjustment was applied. Consequently, *p*-values < 0.01 were considered statistically significant. All statistical analyses were performed with STATA statistical software 12/SE (Stata Corp., College Station, TX, USA).

Estimations of statistical power were obtained with CaTS Power Calculator for Genetic Studies software (Supplementary Tables S2 and S3).

## 3. Results

### 3.1. Analysis of VEGF Genotype, Allele and Carrier Frequencies

The rs833061, rs1570360, rs2010963, rs3025020, and rs3025039 genotype distribution was in HWE. The genotyping success rate was 98.7% for rs833061, 97.9% for rs1570360, 99.1% for rs2010963, 98.6% for rs3025020, and 99.3% for rs3025039. Genotype and allele frequencies were in agreement with the data of the 1000 Genomes Project for Europeans.

Genotype, allele, and carrier frequencies of *VEGF* between the whole cohort of patients with ILD and healthy controls were compared. As shown in Table 2, no statistically significant differences in the frequencies of each *VEGF* polymorphism were found between these groups.

We also assessed the differences in *VEGF* frequencies between the group of patients with IIP and non-IIP patients. In this regard, no statistically significant differences in the genotype, allele, or carrier frequencies of each *VEGF* polymorphism were disclosed between them (Table 3). Likewise, no genetic differences were detected when IIP patients were compared to the group of non-IIP stratified into patients with autoimmune ILDs, hypersensitivity pneumonitis, sarcoidosis, and other ILDs (Supplementary Table S4).

When stratified by sex, no statistically significant differences in the genotype, allele, or carrier frequencies of each *VEGF* polymorphism were disclosed between the whole cohort of ILD patients and healthy controls (Supplementary Table S5). However, the frequency of the minor allele of *VEGF* rs1570360 was increased in women with IIP compared to women with non-IIP (38.0% versus 21.3%, OR = 2.26 [1.27–4.02], *p* = 0.005, Supplementary Table S6).

### 3.2. Haplotype Analysis of VEGF

Haplotype analysis showed five common haplotypes with a frequency greater than 5% both in healthy controls and ILD patients (Table 4). VEGF haplotypes’ frequencies were similar between the whole cohort of patients with ILD and healthy controls and no statistically significant differences were disclosed (Table 4). It was also the case when haplotypes’ frequencies were compared between IIP and non-IIP patients (Table 4).

### 3.3. Association of VEGF Polymorphisms with Pulmonary Function Tests

**Figure 1:**
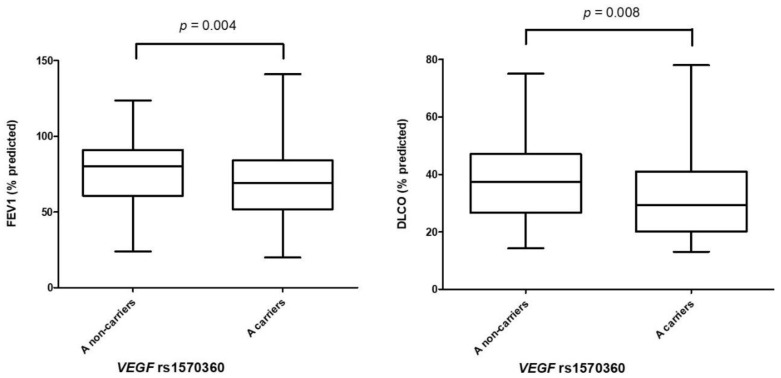
Decrease of forced expiratory volume in one second (FEV1) and diffusing capacity of the lungs for carbon monoxide (DLCO) in patients with idiopathic interstitial pneumonia carrying the minor allele A of *VEGF* rs1570360. Horizontal lines in box plots indicate the mean value of each study group. *p*-values were adjusted by sex, age, smoking history, and packs of cigarettes per year.

Since deterioration of PFTs generally indicates a worse outcome of the ILD, in a further step we determined whether *VEGF* polymorphisms influenced on PFTs in the whole group of patients with IIP and non-IIP. Interestingly, a decrease of FEV1 (% predicted) and DLCO (% predicted) was observed in IIP patients carrying the minor allele A of rs1570360 compared to non-carriers: 69.41 ± 22.34 versus 76.44 ± 21.88 for FEV1 and 32.07 ± 14.34 versus 38.30 ± 15.03 for DLCO, *p* = 0.004 and *p* = 0.008, respectively (Figure 1). No further associations between *VEGF* polymorphisms and PFTs were found in IIP or non-IIP patients (Supplementary Table S7).

### 3.4. Influence of VEGF Polymorphisms on VEGF Serum Levels

Finally, the influence of *VEGF* polymorphisms on VEGF serum levels in IIP and non-IIP patients was assessed. Similar VEGF serum levels were observed in IIP and non-IIP patients, regardless of the *VEGF* polymorphism assessed (Supplementary Table S8).

## 4. Discussion

It is widely known that biomarkers involved in the pathogenesis of ILD are considered as potential targets for therapy [7,8]. In this regard, there is a growing interest in the role of VEGF in the pathogenesis of ILD [18]. Consequently, we aimed to evaluate the role of five functional *VEGF* polymorphisms in the susceptibility and severity of ILD in a large cohort of patients.

In our study, the genotype, allele, and carrier frequencies of rs833061, rs1570360, rs2010963, rs3025020, and rs3025039 were similar between the whole cohort of patients with ILD and healthy controls as well as between IIP and non-IIP patients. Considering that the five polymorphisms assessed are located in relevant regions along the *VEGF* gene, based on our results it is reasonable to think that *VEGF* does not seem to be implicated in ILD. Of note, after conducting a literature review, we noticed that information on the potential role of *VEGF* polymorphisms in the susceptibility and severity of ILD was limited to a single study that only assessed rs2010963 in 60 IPF patients and 60 controls. In this study no association was found between *VEGF* and IPF [33], in keeping with our obtained data. Our results are also in accordance with those reported in other diseases. In particular, no genetic differences of rs699947 (in complete linkage disequilibrium with rs833061, r2 = 0.98, and D′ = 0.99 in European Population) and rs1570360 between patients with COPD and controls were also reported [31]. Likewise, no association with rs1570360 and rs2010963 polymorphisms was observed in rheumatoid arthritis, a chronic inflammatory disease that can be associated with ILD [34,35]. Additionally, haplotype analyses that provide a more comprehensive picture of the implication of a gene in a disease were performed in the present study to uncover hidden signals in the *VEGF* gene. In this regard, there were no significant differences in *VEGF* haplotype frequencies between the whole cohort of patients with ILD and healthy controls as well as between IIP and non-IIP patients, further supporting our findings obtained when polymorphisms were tested individually. Therefore, although VEGF is a potent angiogenic factor associated with pulmonary and chronic inflammatory diseases, our results suggest that it has no influence as a genetic biomarker for the susceptibility of ILD and for the differential diagnosis between the different diseases encompassed under the term ILD. Nevertheless, we disclosed a sex-specific association of the minor allele of *VEGF* rs1570360 with an increased risk of IIP in women. In this line, a gender-specific association with rs1570360 and other *VEGF* polymorphisms has been previously described in women with other non-pulmonary diseases [36,37,38,39]. However, the potential relevance of rs1570360 as a marker of IIP in women should be interpreted with caution considering that the frequency of women with IIP in our population was relatively low. In this sense, we feel that a replication study using an independent cohort of patients with IIP is required to confirm our data.

Regarding the influence of *VEGF* polymorphisms on PFTs, we disclosed, for the first time, a potential association of rs1570360 with a worse lung function in IIP patients. In particular, a significant decrease in FEV1 and DLCO was observed, which may suggest that rs1570360 influences airway function. It is worth mentioning that other *VEGF* polymorphisms were also reported to be associated with an altered lung function in patients with COPD and asthma [23,26]. Further studies on this issue are needed to confirm the role of *VEGF* in lung function.

Finally, we determined whether the *VEGF* polymorphisms assessed may affect VEGF serum levels in ILD patients. However, none of the *VEGF* polymorphisms were associated with VEGF levels in IIP or non-IIP patients. Likewise, Baz–Dávila et al. reported a lack of association between rs833070 (in complete linkage disequilibrium with rs833061, r2 = 0.98, and D′ = 0.99 in European Population) and rs3025020 with VEGF serum levels in Spanish patients with COPD [23]. In addition, rs1570360, rs2010963, and rs3025039 were not related with VEGF serum levels in patients with inflammatory bowel disease [13]. In accordance with these findings, our results support the hypothesis that the regulation of VEGF serum levels may depend on local and systemic inflammatory changes accompanying the disease, rather than on genetic factors, as previously claimed by Almawy et al. [32].

In conclusion, our results do not support a relevant role of *VEGF* in ILD. Nevertheless, they suggest that rs1570360 could be a marker of severity of ILD in women and may indicate a worse outcome in patients with IIP.

## Figures and Tables

**Table 1:** Demographic and clinical characteristics of the 436 patients with ILD included in this study.

Characteristic	Whole Cohort of ILD Patients (*n* = 436)	IIP Patients (*n* = 244)	Non-IIP Patients (*n* = 192)
Sex (men/women), n (%)	294/142 (67.4/32.6)	190/54 (77.9/22.1)	104/88 (54.2/45.8)
Age at the time of the study (years), mean ± SD	61.1 ± 10.2	62.8 ± 9.4	59.0 ± 10.8
Smoking history, n (%)	294 (69.2)	178 (74.2)	116 (62.7)
Packs of cigarettes per year, mean ± SD	30.7 ± 20.2	33.8 ± 20.7	25.8 ± 18.5
Pulmonary function tests			
FVC (% predicted), mean ± SD	76.8 ± 24.1	73.0 ± 22.7	81.7 ± 25.0
FEV1 (% predicted), mean ± SD	73.7 ± 23.6	72.8 ± 22.4	74.8 ± 25.0
DLCO (% predicted), mean ± SD	36.4 ± 15.7	35.2 ± 15.0	37.9 ± 16.6

DLCO: Diffusing capacity of the lungs for carbon monoxide; FEV1: Forced expiratory volume in one second; FVC: Forced vital capacity; IIP: Idiopathic interstitial pneumonia; ILD: Interstitial lung disease; SD: Standard deviation.

**Table 2:** Genotype, allele, and carrier frequencies of *VEGF* polymorphisms in ILD patients and controls included in this study.

*VEGF* Polymorphism	Genotype/Allele/ Carriers	Whole Cohort of ILD Patients % (*n/N*)	Healthy Controls % (*n/N*)	OR [95% CI]	*p*-Value
rs833061	TT	32.6 (142/435)	28.8 (151/524)	Reference	-
	TC	47.6 (207/435)	48.7 (255/524)	0.86 [0.64–1.16]	0.33
	CC	19.8 (86/435)	22.5 (118/524)	0.78 [0.54–1.11]	0.17
	T	56.4 (491/870)	53.1 (557/1048)	Reference	-
	C	43.6 (379/870)	46.9 (491/1048)	0.88 [0.73–1.05]	0.15
	C non-carriers	32.6 (142/435)	28.8 (151/524)	Reference	-
	C carriers	67.4 (293/435)	71.2 (373/524)	0.84 [0.63–1.10]	0.20
rs1570360	GG	53.5 (232/434)	48.3 (250/518)	Reference	-
	GA	38.0 (165/434)	41.9 (217/518)	0.82 [0.63–1.07]	0.15
	AA	8.5 (37/434)	9.8 (51/518)	0.78 [0.49–1.24]	0.29
	G	72.5 (629/868)	69.2 (717/1036)	Reference	-
	A	27.5 (239/868)	30.8 (319/1036)	0.85 [0.70–1.04]	0.12
	A non-carriers	53.5 (232/434)	48.3 (250/518)	Reference	-
	A carriers	46.5 (202/434)	51.7 (268/518)	0.81 [0.63–1.05]	0.11
rs2010963	GG	43.0 (187/435)	43.7 (231/528)	Reference	-
	GC	43.2 (188/435)	46.6 (246/528)	0.94 [0.72–1.24]	0.68
	CC	13.8 (60/435)	9.7 (51/528)	1.45 [0.95–2.21]	0.08
	G	64.6 (562/870)	67.0 (708/1056)	Reference	-
	C	35.4 (308/870)	33.0 (348/1056)	1.11 [0.92–1.35]	0.26
	C non-carriers	43.0 (187/435)	43.7 (231/528)	Reference	-
	C carriers	57.0 (248/435)	56.3 (297/528)	1.03 [0.80–1.33]	0.81
rs3025020	CC	51.9 (225/434)	54.4 (285/524)	Reference	-
	CT	39.6 (172/434)	37.0 (194/524)	1.12 [0.86–1.47]	0.40
	TT	8.5 (37/434)	8.6 (45/524)	1.04 [0.65–1.66]	0.87
	C	71.7 (622/868)	72.9 (764/1048)	Reference	-
	T	28.3 (246/868)	27.1 (284/1048)	1.06 [0.87–1.30]	0.55
	T non-carriers	51.8 (225/434)	54.4 (285/524)	Reference	-
	T carriers	48.2 (209/434)	45.6 (239/524)	1.11 [0.86–1.43]	0.43
rs3025039	CC	74.5 (325/436)	79.0 (418/529)	Reference	-
	CT	22.7 (99/436)	18.9 (100/529)	1.27 [0.93–1.73]	0.13
	TT	2.8 (12/436)	2.1 (11/529)	1.40 [0.61–3.22]	0.42
	C	85.9 (749/872)	88.5 (936/1058)	Reference	-
	T	14.1 (123/872)	11.5 (122/1058)	1.26 [0.96–1.65]	0.09
	T non-carriers	74.5 (325/436)	79.0 (418/529)	Reference	-
	T carriers	25.5 (111/436)	21.0 (111/529)	1.29 [0.95–1.74]	0.10

CI: Confidence interval; ILD: Interstitial lung disease; *N*: Total number of individuals successfully genotyped; OR: Odds ratio; VEGF: Vascular endothelial growth factor.

**Table 3:** Genotype, allele, and carrier frequencies of *VEGF* polymorphisms in IIP and non-IIP patients included in this study.

*VEGF* Polymorphism	Genotype/Allele/ Carriers	IIP Patients % (*n/N*)	Non-IIP Patients % (*n/N*)	OR [95% CI] *	*p*-Value *
rs833061	TT	28.7 (70/244)	37.7 (72/191)	Reference	-
	TC	49.2 (120/244)	45.6 (87/191)	1.32 [0.82–2.11]	0.25
	CC	22.1 (54/244)	16.7 (32/191)	1.73 [0.95–3.16]	0.07
	T	53.3 (260/488)	60.5 (231/382)	Reference	-
	C	46.7 (228/488)	39.5 (151/382)	1.33 [0.99–1.79]	0.06
	C non-carriers	28.7 (70/244)	37.7 (72/191)	Reference	-
	C carriers	71.3 (174/244)	62.3 (119/191)	1.43 [0.92–2.22]	0.12
rs1570360	GG	47.5 (116/244)	61.1 (116/190)	Reference	-
	GA	43.4 (106/244)	31.1 (59/190)	1.79 [1.14–2.81]	0.01
	AA	9.1 (22/244)	7.8 (15/190)	1.50 [0.68–3.31]	0.31
	G	69.3 (338/488)	76.6 (291/380)	Reference	-
	A	30.7 (150/488)	23.4 (89/380)	1.46 [1.04–2.05]	0.03
	A non-carriers	47.5 (116/244)	61.1 (116/190)	Reference	-
	A carriers	52.5 (128/244)	38.9 (74/190)	1.74 [1.14–2.65]	0.01
rs2010963	GG	44.7 (109/244)	40.8 (78/191)	Reference	-
	GC	42.2 (103/244)	44.5 (85/191)	0.80 [0.51–1.25]	0.32
	CC	13.1 (32/244)	14.7 (28/191)	0.66 [0.34–1.25]	0.20
	G	65.8 (321/488)	63.1 (241/382)	Reference	-
	C	34.2 (167/488)	36.9 (141/382)	0.80 [0.59–1.08]	0.15
	C non-carriers	44.7 (109/244)	40.8 (78/191)	Reference	-
	C carriers	55.3 (135/244)	59.2 (113/191)	0.76 [0.50–1.16]	0.20
rs3025020	CC	50.4 (123/244)	53.7 (102/190)	Reference	-
	CT	39.8 (97/244)	39.5 (75/190)	1.13 [0.73–1.76]	0.57
	TT	9.8 (24/244)	6.8 (13/190)	1.57 [0.69–3.54]	0.28
	C	70.3 (343/488)	73.4 (279/380)	Reference	-
	T	29.7 (145/488)	26.6 (101/380)	1.20 [0.86–1.67]	0.28
	T non-carriers	50.4 (123/244)	53.7 (102/190)	Reference	-
	T carriers	49.6 (121/244)	46.3 (88/190)	1.20 [0.79–1.82]	0.40
rs3025039	CC	77.5 (189/244)	70.8 (136/192)	Reference	-
	CT	20.5 (50/244)	25.5 (49/192)	0.63 [0.38–1.04]	0.07
	TT	2.0 (5/244)	3.7 (7/192)	1.09 [0.24–5.02]	0.91
	C	87.7 (428/488)	83.6 (321/384)	Reference	-
	T	12.3 (60/488)	16.4 (63/384)	0.72 [0.47–1.12]	0.14
	T non-carriers	77.5 (189/244)	70.8 (136/192)	Reference	-
	T carriers	22.5 (55/244)	29.2 (56/192)	0.66 [0.41–1.06]	0.09

* OR (95% CI) and *p*-values were adjusted by sex, age, smoking history, and packs of cigarettes per year. CI: confidence interval; IIP: Idiopathic interstitial pneumonia; *N*: Total number of individuals successfully genotyped; OR: Odds ratio; VEGF: Vascular endothelial growth factor.

**Table 4:** Haplotype differences of *VEGF* between the whole group of patients with ILD and healthy controls, and between IIP and non-IIP patients.

	Whole Cohort of ILD Patients versus Controls				IIP versus Non-IIP Patients			
Haplotype *	Frequency in ILD % (*n/N*)	Frequency in Controls % (*n/N*)	OR [95% CI]	*p*-Value	Frequency in IIP % (*n/N*)	Frequency in Non-IIP % (*n/N*)	OR [95% CI] ^‡^	*p*-Value ^‡^
TGGCC	31.4 (271/862)	32.6 (330/1012)	Reference	-	30.7 (150/488)	32.4 (121/374)	Reference	-
TGCCC	10.7 (92/862)	9.8 (99/1012)	1.13 [0.82–1.57]	0.46	10.0 (49/488)	11.5 (43/374)	0.81 [0.48–1.36]	0.42
CAGCC	7.1 (61/862)	9.3 (94/1012)	0.79 [0.55–1.13]	0.20	7.8 (38/488)	6.2 (23/374)	1.11 [0.60–2.05]	0.74
CGGCC	7.5 (65/862)	7.7 (78/1012)	1.01 [0.70–1.46]	0.94	8.6 (42/488)	6.2 (23/374)	1.56 [0.86–2.86]	0.15
CAGTC	8.1 (70/862)	7.6 (77/1012)	1.11 [0.77–1.59]	0.58	9.4 (46/488)	6.4 (24/374)	1.67 [0.92–3.05]	0.09

* The polymorphism order was rs833081, rs1570360, rs2010963, rs3025020, and rs3025039. ^‡^ OR 95% CI and *p*-values were adjusted by sex, age, smoking history, and packs of cigarettes per year. CI: Confidence interval; IIP: Idiopathic interstitial pneumonia; ILD: Interstitial lung disease; N: Total number of individuals successfully genotyped; OR: Odds ratio; VEGF: Vascular endothelial growth factor.

## Data Availability

All data generated or analyzed during this study are included in this published article.

## Supplementary Materials

The following are available online at https://www.mdpi.com/article/10.3390/biomedicines9050458/s1, Table S1: Demographic and clinical characteristics of the 192 non-IIP patients included in this study, Table S2: Statistical power estimation of the analysis of the susceptibility of *VEGF* in ILD according to different allele frequencies and ORs, Table S3: Statistical power estimation of the analysis of the severity of *VEGF* in ILD according to different allele frequencies and ORs, Table S4: Genotype, allele, and carrier frequencies of *VEGF* polymorphisms in patients with IIP, autoimmune ILDs, hypersensitivity pneumonitis, sarcoidosis, and other ILDs, Table S5: Genotype, allele, and carrier frequencies of *VEGF* polymorphisms in the whole cohort of ILD patients and healthy controls, stratified according to sex, Table S6: Genotype, allele, and carrier frequencies of *VEGF* polymorphisms in IIP and non-IIP patients, stratified according to sex, Table S7: Influence of *VEGF* polymorphisms on the pulmonary function tests of IIP and non-IIP patients, Table S8: Influence of *VEGF* polymorphisms on the VEGF serum levels in IIP and non-IIP patients.
