# Supplementary material for: Role of VEGF Polymorphisms in the Susceptibility and Severity of Interstitial Lung Disease
Source: Biomedicines. 2021 Apr 22;9(5):458. doi: 10.3390/biomedicines9050458 (PMC8145193; doi:10.3390/biomedicines9050458)
Supplement: Supplementary file 1 [file biomedicines-09-00458-s001.zip › biomedicines-1185177-supplementary.pdf]

biomedicines

MDPI

Supplementary

Table 1. Demographic and clinical characteristics of the 192 non-IIP patients included in this study.

Characteristic	Autoimmune ILDs (*n* = 108)	Hypersensitivity Pneumonitis (*n* = 47)	Sarcoidosis (*n* = 10)	Other ILDs (*n* = 27)
Sex (men/women), n (%)	57/51 (52.8/47.2)	32/15 (68.1/31.9)	7/3 (70.0/30.0)	8/19 (29.6/70.4)
Age at the time of the study (years), mean ± SD	60.6 ± 9.8	61.2 ± 10.0	53.4 ± 14.2	50.7 ± 10.4
Smoking history, n (%)	65 (64.4)	26 (55.3)	4 (40)	21 (77.8)
Packs of cigarettes per year, mean ± SD	26.5 ± 18.0	28.8 ± 19.4	12.0 ± 12.1	22.3 ± 19.6
Pulmonary function tests				
FVC (% predicted), mean ± SD	82.7 ± 24.7	77.6 ± 24.1	72.0 ± 30.2	88.9 ± 24.4
FEV1 (% predicted), mean ± SD	79.5 ± 24.4	72.3 ± 24.6	57.6 ± 20.3	67.4 ± 26.0
DLCO (% predicted), mean ± SD	37.4 ± 14.8	39.4 ± 21.1	49.7 ± 17.4	30.7 ± 10.9

DLCO: diffusing capacity of the lungs for carbon monoxide; FEV1: forced expiratory volume in one second; FVC: forced vital capacity; IIP: idiopathic interstitial pneumonia; ILD: interstitial lung disease; SD: standard deviation.

Table 2. Statistical power estimation of the analysis of the susceptibility of *VEGF* in ILD according to different allele frequencies and ORs.

Allele Frequency	OR = 1.1	OR = 1.2	OR = 1.3	OR = 1.4	OR = 1.5	OR = 1.6	OR = 1.7	OR = 2.0
10%	10%	23%	44%	65%	82%	92%	97%	100%
20%	13%	37%	66%	87%	96%	99%	100%	100%
30%	16%	46%	77%	94%	99%	100%	100%	100%
40%	18%	42%	70%	96%	99%	100%	100%	100%
50%	18%	52%	82%	96%	99%	100%	100%	100%

Power estimated for a prevalence of interstitial lung disease of 5 cases / 100,000 inhabitants. ILD: interstitial lung disease; OR: odds ratio; VEGF: vascular endothelial growth factor.

Table S3. Statistical power estimation of the analysis of the severity of *VEGF* in ILD according to different allele frequencies and ORs.

Allele Frequency	OR = 1.1	OR = 1.2	OR = 1.3	OR = 1.4	OR = 1.5	OR = 1.6	OR = 1.7	OR = 2.0
10%	7%	13%	23%	36%	49%	63%	74%	94%
20%	9%	20%	36%	55%	71%	84%	92%	99%
30%	10%	24%	44%	65%	81%	91%	96%	100%
40%	11%	26%	48%	69%	84%	93%	97%	100%
50%	11%	27%	48%	69%	84%	93%	97%	100%

Power estimated for a prevalence of idiopathic interstitial pneumonia of 16 cases / 100,000 inhabitants. ILD: interstitial lung disease; OR: odds ratio; VEGF: vascular endothelial growth factor.

Table S4. Genotype, allele and carrier frequencies of *VEGF* polymorphisms in patients with IIP, autoimmune ILDs, hypersensitivity pneumonitis, sarcoidosis and other ILDs.

VEGF Polymorphism	Genotype/Allele/ Carriers	IIP Patients % (*n*/*N*)	Autoimmune ILDs % (*n*/*N*)	Hypersensitivity Pneumonitis % (*n*/*N*)	Sarcoidosis % (*n*/*N*)	Other ILDs* % (*n*/*N*)
rs833061	TT	28.7 (70/244)	42.6 (46/108)	28.3 (13/46)	40.0 (4/10)	33.3 (9/27)
	TC	49.2 (120/244)	39.8 (43/108)	56.5 (26/46)	50.0 (5/10)	48.2 (13/27)
	CC	22.1 (54/244)	17.6 (19/108)	15.2 (7/46)	10.0 (1/10)	18.5 (5/27)
	T	53.3 (260/488)	62.5 (135/216)	56.5 (52/92)	65.0 (13/20)	57.4 (31/54)
	C	46.7 (228/488)	37.5 (81/216)	43.5 (40/92)	35.0 (7/20)	42.6 (23/54)
	C non-carriers	28.7 (70/244)	42.6 (46/108)	28.3 (13/46)	40.0 (4/10)	33.3 (9/27)
	C carriers	71.3 (174/244)	57.4 (162/108)	71.7 (33/46)	60.0 (6/10)	66.7 (18/27)
rs1570360	GG	47.5 (116/244)	60.7 (65/107)	63.1 (29/46)	70.0 (7/10)	55.6 (15/27)
	GA	43.4 (106/244)	29.9 (32/107)	30.4 (14/46)	20.0 (2/10)	40.7 (11/27)
	AA	9.1 (22/244)	9.4 (10/107)	6.5 (3/46)	10.0 (1/10)	3.7 (1/27)
	G	69.3 (338/488)	75.7 (162/214)	78.3 (72/92)	80.0 (16/20)	75.9 (41/54)
	A	30.7 (150/488)	24.3 (52/214)	21.7 (20/92)	20.0 (4/20)	24.1 (13/54)
	A non-carriers	47.5 (116/244)	60.7 (65/107)	63.1 (29/46)	70.0 (7/10)	55.6 (15/27)
	A carriers	52.5 (128/244)	39.3 (42/107)	36.9 (17/46)	30.0 (3/10)	44.4 (12/27)
rs2010963	GG	44.7 (109/244)	39.8 (43/108)	43.5 (20/46)	30.0 (3/10)	44.4 (12/27)
	GC	42.2 (103/244)	41.7 (45/108)	50.0 (23/46)	40.0 (4/10)	48.2 (13/27)
	CC	13.1 (32/244)	18.5 (20/108)	6.5 (3/46)	30.0 (3/10)	7.4 (2/27)
	G	65.8 (321/488)	60.7 (131/216)	68.5 (63/92)	50.0 (10/20)	68.5 (37/54)
	C	34.2 (167/488)	39.3 (85/216)	31.5 (29/92)	50.0 (10/20)	31.5 (17/54)
	C non-carriers	44.7 (109/244)	39.8 (43/108)	43.5 (20/46)	30.0 (3/10)	44.4 (12/27)
	C carriers	55.3 (135/244)	60.2 (65/108)	56.5 (26/46)	70.0 (7/10)	55.6 (15/27)
rs3025020	CC	50.4 (123/244)	55.1 (59/107)	63.0 (29/46)	30.0 (3/10)	40.7 (11/27)
	CT	39.8 (97/244)	40.2 (43/107)	30.4 (14/46)	50.0 (5/10)	48.2 (13/27)
	TT	9.8 (24/244)	4.7 (5/107)	6.6 (3/46)	20.0 (2/10)	11.1 (3/27)
	C	70.3 (343/488)	75.2 (161/214)	78.3 (72/92)	55.0 (11/20)	64.8 (35/54)
	T	29.7 (145/488)	24.8 (53/214)	21.7 (20/92)	45.0 (9/20)	35.2 (19/54)
	T non-carriers	50.4 (123/244)	55.1 (59/107)	63.0 (29/46)	30.0 (3/10)	40.7 (11/27)
	T carriers	49.6 (121/244)	44.9 (48/107)	37.0 (17/46)	70.0 (7/10)	59.3 (16/27)
**rs3025039**	CC	77.5 (189/244)	68.5 (74/108)	72.4 (34/47)	80.0 (8/10)	74.1 (20/27)
	CT	20.5 (50/244)	26.9 (29/108)	25.5 (12/47)	10.0 (1/10)	25.9 (7/27)
	TT	2.0 (5/244)	4.6 (5/108)	2.1 (1/47)	10.0 (1/10)	0
	C	87.7 (428/488)	81.9 (177/216)	85.1 (80/94)	85.0 (17/20)	87.0 (47/54)
	T	12.3 (60/488)	18.1 (39/216)	14.9 (14/94)	15.0 (3/20)	13.0 (7/54)
	T non-carriers	77.5 (189/244)	68.5 (74/108)	72.4 (34/47)	80.0 (8/10)	74.1 (20/27)
	T carriers	22.5 (55/244)	31.5 (34/108)	27.6 (13/47)	20.0 (2/10)	25.9 (7/27)

IIP: idiopathic interstitial pneumonia; ILD: interstitial lung disease; N: total number of individuals successfully genotyped; VEGF: vascular endothelial growth. *Other ILDs include patients with lymphangioleiomyomatosis, Langerhans' cell histiocytosis and drug-associated ILD. All the genotype, allele and carrier frequencies did not show statistically significant differences

Table S5. Genotype, allele and carrier frequencies of *VEGF* polymorphisms in the whole cohort of ILD patients and healthy controls, stratified according to sex.

VEGF Polymorphism	Genotype/Allele/Carriers	MEN				WOMEN			
		ILD patients % (*n*/*N*)	Controls % (*n*/*N*)	OR [95% CI]	p value	ILD patients % (*n*/*N*)	Controls % (*n*/*N*)	OR [95% CI]	*p* value
rs833061	TT	31.7 (93/293)	31.1 (79/254)	Reference	-	34.5 (49/142)	26.7 (72/270)	Reference	-
	TC	49.2 (144/293)	45.7 (116/254)	1.05 [0.72-1.55]	0.79	44.4 (63/142)	51.5 (139/270)	0.67 [0.42-1.06]	0.09
	CC	19.1 (56/293)	23.2 (59/254)	0.81 [0.50-1.29]	0.37	21.1 (30/142)	21.9 (59/270)	0.75 [0.42-1.32]	0.32
	T	56.3 (330/586)	53.9 (274/508)	Reference	-	56.7 (161/284)	52.4 (283/540)	Reference	-
	C	43.7 (256/586)	46.1 (234/508)	0.91 [0.72-1.15]	0.43	43.3 (123/284)	47.6 (257/540)	0.84 [0.63-1.12]	0.24
	C non-carriers	31.7 (93/293)	31.1 (79/254)	Reference	-	34.5 (49/142)	26.7 (72/270)	Reference	-
	C carriers	68.3 (200/293)	68.9 (175/254)	0.97 [0.68-1.39]	0.87	65.5 (93/142)	73.3 (198/270)	0.69 [0.45-1.07]	0.10
rs1570360	GG	54.3 (159/293)	48.0 (119/248)	Reference	-	51.8 (73/141)	48.5 (131/270)	Reference	-
	GA	36.5 (107/293)	41.9 (104/248)	0.77 [0.54-1.10]	0.15	41.1 (58/141)	41.9 (113/270)	0.92 [0.60-1.41]	0.71
	AA	9.2 (27/293)	10.1 (25/248)	0.81 [0.45-1.46]	0.48	7.1 (10/141)	9.6 (26/270)	0.69 [0.32-1.51]	0.35
	G	72.5 (425/586)	68.9 (342/496)	Reference	-	72.3 (204/282)	69.4 (375/540)	Reference	-
	A	27.5 (161/586)	31.1 (154/496)	0.84 [0.65-1.09]	0.20	27.7 (78/282)	30.6 (165/540)	0.87 [0.63-1.20]	0.39
	A non-carriers	54.3 (159/293)	48.0 (119/248)	Reference	-	51.8 (73/141)	48.5 (131/270)	Reference	-
	A carriers	45.7 (134/293)	52.0 (129/248)	0.78 [0.55-1.09]	0.15	48.2 (68/141)	51.5 (139/270)	0.88 [0.58-1.32]	0.53
rs2010963	GG	42.3 (124/293)	42.8 (110/257)	Reference	-	44.4 (63/142)	44.6 (121/271)	Reference	-
	GC	44.0 (129/293)	45.1 (116/257)	0.99 [0.69-1.41]	0.94	41.5 (59/142)	48.0 (130/271)	0.87 [0.57-1.34]	0.53
	CC	13.7 (40/293)	12.1 (31/257)	1.14 [0.67-1.95]	0.62	14.1 (20/142)	7.4 (20/271)	1.92 [0.96-3.83]	0.06
	G	64.3 (337/586)	65.4 (336/514)	Reference	-	65.1 (185/284)	68.6 (372/542)	Reference	-
	C	35.7 (209/586)	34.6 (178/514)	1.05 [0.82-1.34]	0.72	34.9 (99/284)	31.4 (170/542)	1.17 [0.86-1.59]	0.31
	C non-carriers	42.3 (124/293)	42.8 (110/257)	Reference	-	44.4 (63/142)	44.6 (121/271)	Reference	-
	C carriers	57.7 (169/293)	57.2 (147/257)	1.02 [0.73-1.43]	0.91	55.6 (79/142)	55.4 (150/271)	1.01 [0.67-1.52]	0.96

rs3025020	CC	52.2 (153/293)	53.3 (136/255)	Reference	-	51.1 (72/141)	55.4 (149/269)	Reference	-
	CT	38.9 (114/293)	37.3 (95/255)	1.07 [0.75-1.52]	0.72	41.1 (58/141)	36.8 (99/269)	1.21 [0.79-1.86]	0.38
	TT	8.9 (26/293)	9.4 (24/255)	0.96 [0.53-1.76]	0.90	7.8 (11/141)	7.8 (21/269)	1.08 [0.50-2.37]	0.84
	C	71.7 (420/586)	72.0 (367/510)	Reference	-	71.6 (202/282)	73.8 (397/538)	Reference	-
	T	28.3 (166/586)	28.0 (143/510)	1.01 [0.78-1.32]	0.92	28.4 (80/282)	26.2 (141/538)	1.12 [0.81-1.54]	0.51
	T non-carriers	52.2 (153/293)	53.3 (136/255)	Reference	-	51.1 (72/141)	55.4 (149/269)	Reference	-
	T carriers	47.8 (140/293)	46.7 (119/255)	1.05 [0.75-1.46]	0.79	48.9 (69/141)	44.6 (120/269)	1.19 [0.79-1.79]	0.40
rs3025039	CC	75.5 (222/294)	76.3 (196/257)	Reference	-	72.6 (103/142)	81.6 (222/272)	Reference	-
	CT	23.1 (68/294)	21.8 (56/257)	1.07 [0.72-1.60]	0.74	21.8 (31/142)	16.2 (44/272)	1.52 [0.91-2.54]	0.11
	TT	1.4 (4/294)	1.9 (5/257)	0.71 [0.19-2.67]	0.61	5.6 (8/142)	2.2 (6/272)	2.87 [0.97-8.50]	0.06
	C	87.1 (512/588)	87.2 (448/514)	Reference	-	83.5 (237/284)	89.7 (488/544)	Reference	-
	T	12.9 (76/588)	12.8 (66/514)	1.01 [0.71-1.43]	0.97	16.5 (47/284)	10.3 (56/544)	1.73 [1.14-2.62]	0.01
	T non-carriers	75.5 (222/294)	76.3 (196/257)	Reference	-	72.6 (103/142)	81.6 (222/272)	Reference	-
	T carriers	24.5 (72/294)	23.7 (61/257)	1.04 [0.70-1.54]	0.84	27.4 (39/142)	18.4 (50/272)	1.68 [1.04-2.72]	0.03

CI: confidence interval; ILD: interstitial lung disease; N: total number of individuals successfully genotyped; OR: odds ratio; VEGF: vascular endothelial growth factor.

Table S6. Genotype, allele and carrier frequencies of *VEGF* polymorphisms in IIP and non-IIP patients, stratified according to sex.

***VEGF* Polymorphism**	**Genotype/Allele/Carriers**	**MEN**				**WOMEN**			
		**IIP Patients % (*n*/*N*)**	**Non-IIP Patients % (*n*/*N*)**	**OR [95% CI]***	***p* value***	**IIP Patients % (*n*/*N*)**	**Non-IIP Patients % (*n*/*N*)**	**OR [95% CI]***	***p* value***
**rs833061**	TT	30.5 (58/190)	34.0 (35/103)	Reference	-	22.2 (12/54)	42.0 (37/88)	Reference	-
	TC	49.5 (94/190)	48.5 (50/103)	1.01 [0.57-1.81]	0.97	48.2 (26/54)	42.0 (37/88)	2.18 [0.90-5.26]	0.08
	CC	20.0 (38/190)	17.5 (18/103)	1.31 [0.62-2.78]	0.48	29.6 (16/54)	16.0 (14/88)	3.26 [1.14-9.32]	0.03
	T	55.3 (210/380)	58.3 (120/206)	Reference	-	46.3 (50/108)	63.1 (111/176)	Reference	-
	C	44.7 (170/380)	41.7 (86/206)	1.13 [0.78-1.62]	0.52	53.7 (58/108)	36.9 (65/176)	1.89 [1.12-3.18]	0.02

	C non-carriers	30.5 (58/190)	34.0 (35/103)	Reference	-	22.2 (12/54)	42.0 (37/88)	Reference	-
	C carriers	69.5 (132/190)	66.0 (68/103)	1.09 [0.63-1.88]	0.76	77.8 (42/54)	58.0 (51/88)	2.48 [1.09-5.67]	0.03
**rs1570360**	GG	50.5 (96/190)	61.2 (63/103)	Reference	-	37.0 (20/54)	60.9 (53/87)	Reference	-
	GA	41.6 (79/190)	27.2 (28/103)	1.63 [0.92-2.87]	0.09	50.0 (27/54)	35.6 (31/87)	2.11 [0.97-4.60]	0.06
	AA	7.9 (15/190)	11.6 (12/103)	0.88 [0.36-2.13]	0.77	13.0 (7/54)	3.5 (3/87)	8.88 [1.53-50.51]	0.02
	G	71.3 (271/380)	74.8 (154/206)	Reference	-	62.0 (67/108)	78.7 (137/174)	Reference	-
	A	26.7 (109/380)	25.2 (52/206)	1.16 [0.77-1.76]	0.48	**38.0 (41/108)**	**21.3 (37/174)**	**2.26 [1.27-4.02]**	**0.005**
	A non-carriers	50.5 (96/190)	61.2 (63/103)	Reference	-	37.0 (20/54)	60.9 (53/87)	Reference	-
	A carriers	49.5 (94/190)	38.8 (40/103)	1.42 [0.84-2.39]	0.19	63.0 (34/54)	39.1 (34/87)	2.52 [1.19-5.34]	0.02
**rs2010963**	GG	43.2 (82/190)	40.8 (42/103)	Reference	-	50.0 (27/54)	40.9 (36/88)	Reference	-
	GC	43.2 (82/190)	45.6 (47/103)	0.79 [0.45-1.36]	0.39	38.9 (21/54)	43.2 (38/88)	0.78 [0.35-1.72]	0.54
	CC	13.6 (26/190)	13.6 (14/103)	0.71 [0.32-1.58]	0.40	11.1 (6/54)	15.9 (14/88)	0.44 [0.13-1.48]	0.18
	G	64.7 (246/380)	63.6 (131/206)	Reference	-	69.4 (75/108)	62.5 (110/176)	Reference	-
	C	35.3 (134/380)	36.4 (75/206)	0.82 [0.57-1.20]	0.31	30.6 (33/108)	37.5 (66/176)	0.68 [0.39-1.19]	0.18
	C non-carriers	43.2 (82/190)	40.8 (42/103)	Reference	-	50.0 (27/54)	40.9 (36/88)	Reference	-
	C carriers	56.8 (108/190)	59.2 (61/103)	0.77 [0.46-1.29]	0.32	50.0 (27/54)	59.1 (52/88)	0.68 [0.33-1.44]	0.32
**rs3025020**	CC	52.1 (99/190)	52.4 (54/103)	Reference	-	44.4 (24/54)	55.2 (48/87)	Reference	-
	CT	37.9 (72/190)	40.8 (42/103)	0.92 [0.54-1.57]	0.75	46.3 (25/54)	37.9 (33/87)	1.71 [0.78-3.72]	0.18
	TT	10.0 (19/190)	6.8 (7/103)	1.82 [0.62-5.35]	0.27	9.3 (5/54)	6.9 (6/87)	1.39 [0.34-5.67]	0.64
	C	71.1 (270/380)	72.8 (150/206)	Reference	-	67.6 (73/108)	74.1 (129/174)	Reference	-
	T	28.9 (110/380)	27.2 (56/206)	1.13 [0.75-1.69]	0.57	32.4 (35/108)	25.9 (45/174)	1.37 [0.78-2.43]	0.28
	T non-carriers	52.1 (99/190)	52.4 (54/103)	Reference	-	44.4 (24/54)	55.2 (48/87)	Reference	-
	T carriers	47.9 (91/190)	47.6 (49/103)	1.02 [0.61-1.70]	0.94	55.6 (30/54)	44.8 (39/87)	1.65 [0.78-3.47]	0.19
**rs3025039**	CC	77.8 (148/190)	71.2 (74/104)	Reference	-	75.9 (41/54)	70.4 (62/88)	Reference	-
	CT	21.1 (40/190)	26.9 (28/104)	0.59 [0.32-1.07]	0.08	18.5 (10/54)	23.9 (21/88)	0.81 [0.33-2.01]	0.65

TT	1.1 (2/190)	1.9 (2/104)	0.48 [0.03-8.33]	0.61	5.6 (3/54)	5.7 (5/88)	1.47 [0.25-8.42]	0.67
C	88.4 (336/380)	84.6 (176/208)	Reference	-	85.2 (92/108)	82.4 (145/176)	Reference	-
T	11.6 (44/380)	15.4 (32/208)	0.62 [0.37-1.06]	0.08	14.8 (16/108)	17.6 (31/176)	1.00 [0.49-2.04]	0.99
T non-carriers	77.8 (148/190)	71.2 (74/104)	Reference	-	75.9 (41/54)	70.4 (62/88)	Reference	-
T carriers	22.2 (42/190)	28.9 (30/104)	0.58 [0.32-1.05]	0.07	24.1 (13/54)	29.6 (26/88)	0.90 [0.39-2.08]	0.81

*OR [95% CI] and p values were adjusted by age, smoking history and packs of cigarettes per year.
Significant results are highlighted in **bold.**
CI: confidence interval; IIP: idiopathic interstitial pneumonia; N: total number of individuals successfully genotyped; OR: odds ratio; VEGF: vascular endothelial growth factor.

Table S7. Influence of *VEGF* polymorphisms on the pulmonary function tests of IIP and non-IIP patients.

Pulmonary function tests	*VEGF* polymorphism	Carriers	IIP patients	Non-IIP patients
FVC (% predicted), mean ± SD	**rs833061**	C non-carriers	76.15 ± 22.54	82.46 ± 26.88
		C carriers	71.75 ± 22.67	80.92 ± 23.54
	**rs1570360**	A non-carriers	76.41 ± 22.28	80.11 ± 25.37
		A carriers	69.92 ± 22.67	83.50 ± 23.99
	**rs2010963**	C non-carriers	70.29 ± 21.41	83.11 ± 24.94
		C carriers	75.19 ± 23.49	80.90 ± 25.11
	**rs3025020**	T non-carriers	73.53 ± 21.06	83.56 ± 26.16
		T carriers	72.47 ± 24.28	79.70 ± 23.67
	**rs3025039**	T non-carriers	72.05 ± 22.61	81.42 ± 23.45
		T carriers	76.24 ± 22.81	82.52 ± 28.50
FEV1 (% predicted), mean ± SD	**rs833061**	C non-carriers	75.20 ± 20.69	78.21 ± 26.21
		C carriers	71.78 ± 22.97	72.32 ± 23.87
	**rs1570360**	A non-carriers	**76.44 ± 21.88***	75.19 ± 24.67
		A carriers	**69.41 ± 22.34***	73.15 ± 25.25
	**rs2010963**	C non-carriers	69.37 ± 21.33	75.86 ± 24.56
		C carriers	75.47 ± 22.87	74.15 ± 25.56
	**rs3025020**	T non-carriers	73.12 ± 21.18	78.44 ± 25.70
		T carriers	72.37 ± 23.58	70.50 ± 23.95
	**rs3025039**	T non-carriers	72.23 ± 23.15	74.56 ± 24.11
		T carriers	74.52 ± 19.50	75.44 ± 27.41
DLCO (% predicted), mean ± SD	**rs833061**	C non-carriers	37.39 ± 14.56	40.00 ± 18.12
		C carriers	34.22 ± 15.12	36.08 ± 14.83
	**rs1570360**	A non-carriers	**38.30 ± 15.03‡**	37.58 ± 17.16
		A carriers	**32.07 ± 14.34‡**	36.64 ± 13.81
	**rs2010963**	C non-carriers	33.93 ± 14.24	37.50 ± 18.08
		C carriers	36.16 ± 15.51	38.46 ± 15.30
	**rs3025020**	T non-carriers	36.50 ± 15.74	38.08 ± 17.45
		T carriers	34.07 ± 14.27	37.58 ± 15.79
	**rs3025039**	T non-carriers	36.09 ± 15.73	38.21 ± 16.82
		T carriers	32.12 ± 11.66	36.96 ± 16.15

DLCO: diffusing capacity of the lungs for carbon monoxide; FEV1: forced expiratory volume in one second; FVC: forced vital capacity; IIP: idiopathic interstitial pneumonia; SD: standard deviation; VEGF: vascular endothelial growth factor.

PFTs values are shown as mean ± SD.

*Statistically significant differences obtained after adjustment by sex, age, smoking history and packs of cigarettes per year ($p = 0.004$).

‡Statistically significant differences obtained after adjustment by sex, age, smoking history and packs of cigarettes per year ($p = 0.008$).

Table S8. Influence of *VEGF* polymorphisms on the VEGF serum levels in IIP and non-IIP patients.

		VEGF Serum Levels, Mean ± SD, pg/mL	
VEGF polymorphism	**Carriers**	**IIP Patients**	**Non-IIP Patients**
rs833061	C non-carriers	95.87 ± 115.43	103.83 ± 84.32
	C carriers	97.60 ± 117.56	123.85 ± 131.15
rs1570360	A non-carriers	91.55 ± 104.41	105.11 ± 80.03
	A carriers	102.24 ± 127.25	135.81 ± 158.14
rs2010963	C non-carriers	91.04 ± 123.18	117.98 ± 112.54
	C carriers	101.92 ± 111.51	114.94 ± 118.09
rs3025020	T non-carriers	94.39 ± 105.82	118.38 ± 106.02
	T carriers	99.46 ± 125.80	114.70 ± 128.09
rs3025039	T non-carriers	91.00 ± 113.76	120.62 ± 130.60
	T carriers	124.77 ± 127.10	105.32 ± 71.53

IIP: idiopathic interstitial pneumonia; SD: standard deviation; VEGF: vascular endothelial growth factor.

Comparisons between non-carriers and carriers of each polymorphism did not show statistically significant differences after adjustment by sex, age, smoking history and packs of cigarettes per year (p >0.01).
